# Psychometric evaluation of the Pittsburgh Sleep Quality Index among South African women participating in the Bukhali trial: Healthy Life Trajectories Initiative

**DOI:** 10.1007/s44470-026-00146-z

**Published:** 2026-08-03

**Authors:** Stephanie Alcock, Johanna Beukes, Claire Hart, Karine Scheuermaier, Stephen J. Lye, Shane A. Norris

**Affiliations:** 1 SAMRC/Wits Ageing African Adult Research Unit, Department of Paediatrics, School of Clinical Medicine, Faculty of Health Sciences, University of the Witwatersrand, Johannesburg, South Africa; 2 Wits Sleep Lab, Brain Function Research Group, Department of Physiology, School of Biomedical Sciences, Faculty of Health Sciences, University of the Witwatersrand, Johannesburg, South Africa; 3 Lunenfeld-Tanenbaum Research Institute, Toronto, ON Canada; 4 School of Human Development and Health, Faculty of Medicine, University of Southampton, Southampton, UK

**Keywords:** Pittsburgh Sleep Quality Index, Sleep quality, Psychometric properties, Confirmatory factor analysis, South Africa

## Abstract

**Purpose:**

We examined the psychometric properties of the Pittsburgh Sleep Quality Index (PSQI) in a large sample of young women in Soweto, South Africa, to assess its reliability and structural validity in this context.

**Methods:**

Data were collected from 7182 women enrolled in the Bukhali randomized controlled trial, part of the Healthy Life Trajectories Initiative (HeLTI). Sociodemographic information and PSQI data were collected through interviewer-administered surveys. Internal consistency was assessed using Cronbach’s alpha, McDonald’s omega and item-level correlations. Confirmatory factor analysis (CFA) evaluated the original one-factor and established two- and three-factor multidimensional models. Model fit was examined using Root Mean Square Error of Approximation (RMSEA), Comparative Fit Index (CFI), and Tucker–Lewis Index (TLI).

**Results:**

Most women (57.4%) reported good sleep quality (PSQI ≤ 5). Poor sleep quality was associated with higher household socioeconomic status (assets score), higher education, and single relationship status. Average sleep was 7.8 hours, characterized by prolonged onset latency and high fragmentation, suggesting that continuity disruptions rather than opportunity shortage drove poor sleep. The PSQI had modest internal consistency (α = .57; ω = .57; item–total *r* = .33 – .65). The one-factor model demonstrated poor fit (χ^2^(14) = 1677.70; CFI = .65; TLI = .48; RMSEA = .13) whereas fit was good for the two-factor (χ^2^(13) = 291.46; CFI = .94; TLI = .91; RMSEA = .05) and three-factor models (χ^2^(11) = 285.21; CFI = .94; TLI = .89; RMSEA = .06).

**Conclusion:**

The PSQI showed a multidimensional structure in this population. While both multidimensional models performed well, the two-factor model showed slightly better fit and is recommended for use in this population. Interpreting PSQI components rather than the global score offers more meaningful insight into sleep quality and highlights the need for context-specific psychometric validation in diverse settings.

**Brief Summary:**

**Current Knowledge/Study Rationale:**

The Pittsburgh Sleep Quality Index is widely used, but prior evidence shows its reliability and factor structure vary across populations, settings, and sex. Young women in under-resourced urban South African settings may experience sleep disruption shaped by psychosocial stress, safety concerns, caregiving demands, and environmental factors, yet the PSQI had not been evaluated in this population.

**Study Impact:**

In this large Soweto cohort, sleep problems appeared driven more by fragmented sleep and continuity disruptions than by insufficient sleep opportunity. The PSQI performed better as a multidimensional measure, particularly a parsimonious two-factor structure, than as a single global score. The findings support using component- or factor-informed PSQI interpretation and highlight the need for context-specific validation of sleep measures in diverse populations.

**Supplementary Information:**

The online version contains supplementary material available at 10.1007/s44470-026-00146-z.

## Introduction

Sleep is a key determinant of health and well-being. Approximately 16% of the global population is affected by poor sleep quality [1], which is an emerging public health issue due to its association with increased risk of non-communicable disease (NCD) [2, 3], mental health conditions [4] and adverse socioeconomic outcomes [5]. In low- and middle-income countries (LMICs), poor sleep disproportionately affects low socioeconomic status (SES) groups, particularly women who encounter various vulnerabilities such as low income, informal or insecure employment, high caregiving demands, food insecurity, and exposure to gender-based violence and chronic stress [6].

Reliable sleep quality measures are thus essential across diverse populations. The Pittsburgh Sleep Quality Index (PSQI) is a commonly used self-report instrument that assesses sleep quality over a one-month interval [7]. It comprises 19 items grouped into seven components (subjective sleep quality, sleep latency, sleep duration, habitual sleep efficiency, sleep disturbances, use of sleep medication, and daytime dysfunction) and is rated from 0 to 3. Components are combined to produce a global score ranging from 0 to 21, with higher scores indicating poorer quality sleep.

Research guided by the Consensus-based Standards for the selection of health status Measurement Instruments (COSMIN) standards [8] shows acceptable internal consistency (Cronbach’s *α* = 0.70–0.83) across clinical and non-clinical populations [9] and strong test–retest reliability (*r* ≈ 0.87) over short intervals [10], however, estimates may vary by study design and retest duration [9]. Additionally, the PSQI demonstrates good content validity, based on domain coverage [11], construct validity, through moderate correlations with related constructs (i.e., anxiety and depression) and weak associations with unrelated variables [9, 12–14], as well as criterion validity due to its ability to distinguish disordered from healthy sleep [9] and its associations with other self-reported measures, such as the Epworth Sleepiness Scale (ESS) [11].

However, challenges in the PSQI have been identified, including low factor loadings for the *use of sleep medication* and *daytime dysfunction* components [9, 10], often attributed to low prevalence or contextual variability, weaker associations with objective measures, such as polysomnography, reflecting its subjective nature [15], and mixed findings from confirmatory factor analysis (CFA), which report one- [16, 17], two- [18–20] and three-factor [21, 22] models that vary across context, population and sex [23–26],

Evidence from South Africa also suggests that PSQI performance is population dependent. Among 612 male long-haul truck drivers, poor internal consistency (*α* = 0.42) and a three-factor structure that explained only 19.6% of the variance, likely due to irregular schedules impairing recall [27]. Conversely, acceptable internal consistency (*α* = 0.78) and good construct validity emerged in 139 HIV-positive adults at Chris Hani Baragwanath Hospital [28].

While these findings demonstrate that the PSQI is context-specific, no study has examined the PSQI in young, low-SES women from urban settings, where vulnerability to poor sleep may be greater due to chronic stress, environmental noise, and safety concerns. Therefore, we evaluated the psychometric properties of the PSQI, including internal consistency, construct validity, and factorial structure, in a large sample of young women in Soweto, South Africa, thereby providing preliminary evidence regarding its appropriateness for use in this context.

## Material and methods

### Population and setting

This study was a secondary analysis of the data derived from the Bukhali randomized controlled trial, which forms part of the Healthy Life Trajectories Initiative (HeLTI). The primary aim of HeLTI is to evaluate the impact of comprehensive health initiatives on the well-being of young women aged 18–28 years [29]. The HeLTI data were obtained from individuals residing in the Soweto region, a densely populated urban area of Johannesburg that predominantly comprises a low- to middle-income and multilingual population. A community-based survey of approximately 30,000 households was used for recruitment [29]. Inclusion in the study was conditional on women being non-pregnant and with no prior diagnoses of cancer, Type I diabetes, or epilepsy. Baseline data were collected from 7735 women, including sociodemographics and the PSQI. After excluding those with any missing data or impossible values, the analytic sample for this sub-study comprised 7182 women.

### Measures

#### Sociodemographic factors

Participants’ age, education, relationship status and socioeconomic status (SES) were recorded. Socioeconomic status was determined using a household asset score from a list of 13 common assets (electricity, fridge, stove, vacuum cleaner, washing machine, satellite TV, DVD player, car, TV, landline telephone, cell phone, computer/laptop/tablet, and internet access). This measure has been shown to be a key indicator of household economic status and sensitive to change over time [30, 31]. Scores ranged from 0 to 13, with higher scores indicating higher SES.

#### Pittsburgh Sleep Quality Index

The PSQI is a self-report measure of sleep quality comprising 19 items that are combined to create seven component scores producing a global score (0–21), with higher values indicating poorer sleep quality. Consistent with standard practice, a global score > 5 was used to classify poor sleep quality [7]. The original single-factor model was compared with the multidimensional subscale models proposed by Jia et al. [32]. The two-factor model distinguishes between sleep efficiency (sleep duration and habitual sleep efficiency) and sleep latency (subjective sleep quality, sleep latency, sleep disturbances, use of sleep medication, and daytime dysfunction). The three-factor model further refined this structure into sleep efficiency (sleep duration and habitual sleep efficiency), sleep latency (sleep latency and use of sleep medication), and sleep quality (subjective sleep quality, sleep disturbances, and daytime dysfunction).

#### Data analysis

All analyses were conducted using Stata® v19 (StataCorp, College Station, TX, USA). Data cleaning and screening were conducted prior to analysis. Bedtime and wake times were converted to minutes since midnight. To correctly calculate the interval between them, adjustments were made for midnight crossing. Specifically, when the reported wake time occurred on the following clock day relative to bedtime (e.g., bed at 23:00 and wake at 06:00), 24 h were added to the wake time. Time in bed was then compared with reported sleep duration, and cases were identified as impossible where sleep duration exceeded time in bed, time in bed exceeded 20 h, sleep duration was 0 h, or sleep latency exceeded 180 min, and were removed prior to analysis. Although missingness across components was low (< 5%), Little’s test for missing completely at random (MCAR) indicated that the data were not missing completely at random (*p* < 0.001).

 Assessment of the distributional properties of PSQI components using Doornik–Hansen’s test demonstrated deviations from univariate and multivariate normality (*p* < 0.001). Therefore, confirmatory factor analysis (CFA) models were estimated using maximum likelihood with missing values (MLMV), which is a full-information maximum likelihood approach that uses all available data and provides valid inference under MCAR and missing at random (MAR) assumptions [33]. Despite non-normality, the large sample size and relative robustness of maximum likelihood estimation under moderate non-normality justified its use [34].

Descriptive statistics summarized sociodemographic characteristics, with continuous variables summarized as means and standard deviations (SD) and categorical variables as frequencies (%). Chi-square (*χ*^2^; categorical) and independent-samples *t*-tests were used to assess group differences.

Internal consistency reliability for the PSQI was examined using both Cronbach’s alpha (*α*) and McDonald’s omega (*ω*) coefficients, which is preferred in cases where scales include a greater number of items (≥ 5 items) [35]. Although values of ≥ 0.70 are desirable [36], lower thresholds (≥ 0.5) are also considered acceptable [37]. Item-test correlations, inter-item correlations, and alpha values if items were deleted were assessed to evaluate each component’s contribution to the total score.

Confirmatory factor analysis (CFA) were used to test and compare three PSQI measurement models. Goodness-of-fit was evaluated using multiple indicators, including chi-square (χ^2^), root mean square error of approximation (RMSEA), comparative fit index (CFI), and Tucker–Lewis index (TLI). Model fit was assessed using frequently cited cutoff criteria, with non-significant χ^2^ indicating acceptable fit, CFI and TLI values ≥ 0.90 were interpreted as acceptable, and values ≥ 0.95 as indicative of good fit, and RMSEA values ≤ 0.06 reflecting good fit [38]. While *χ*^2^ was used as an overall measure of model fit, its significance was interpreted cautiously given that it is sensitive to sample size [39]. Therefore, model fit was evaluated primarily using CFI, TLI, and RMSEA. Statistical significance was set at *p* < 0.05 (two-tailed). Modification indices were not examined, as the aim was to evaluate pre-specified models.

#### Ethical considerations

Ethical approval was obtained from the Human Research Ethics Committee (Medical) of the University of the Witwatersrand (ethical clearance number; M1811111). The trial is registered with the Pan African Clinical Trials Registry (https://pactr.samrc.ac.za identifier: PACTR201903750173871). Written informed consent was obtained from all participants.

## Results

Table 1 presents the descriptive sleep characteristics, while the Supplementary Material (Table S1) provides the full descriptive statistics at the item and component levels. Participants reported a relatively early median bedtime (22:00, IQR: 21:00–22:30) and wake time (07:00, IQR: 06:00–08:00, corrected for clock time). Participants spent an average of 9.5 h in bed (SD = 2.18) but reported sleeping only 7.8 h (SD = 1.88). Median sleep onset latency was 20 min (IQR: 10–30), and sleep fragmentation scores indicated frequent nocturnal disturbances. Longer time in bed was moderately associated with longer sleep duration (*r* = 0.584, *p* < 0.001), suggesting that increased sleep opportunity translated into more sleep. However, greater sleep fragmentation was weakly but significantly associated with shorter sleep duration (*r* = − 0.11, *p* < 0.001) and shorter time in bed (*r* = − 0.01, *p* < 0.001). This pattern suggests that although participants had sufficient opportunity to sleep, nocturnal disruptions reduced the amount of sleep obtained, indicating that poor sleep quality was primarily driven by sleep continuity problems rather than insufficient sleep opportunity.

**Table 1:** Descriptive data of sleep timing, time in bed, sleep duration, and sleep onset latency (*n* = 7182)

Variable	Mean (SD)	Median (IQR)	Skewness	Kurtosis	1	2	3	4	5
1. Bedtime (24-h clock, corrected)	21.63 (2)	22 (21–22.5)	− 2.29	17.1					
2. Wake time (24-h clock)	7.15 (1.7)	7 (6–8)	−.45	12.11	.319***				
3. Time in bed (hours)	9.52 (2.18)	9.25 (8–11)	1.1	7.26	−.676***	.454***			
4. Sleep duration (hours)	7.81 (1.88)	8 (7–9)	.09	3.36	−.273***	.427***	.584***		
5. Sleep onset latency (minutes)	25.31 (24.46)	20 (10–30)	2.47	12.68	−.011	.023*	.028*	−.064***	
6. Sleep fragmentation	6.87 (4.63)	6 (3–10)	.62	2.98	−.005	−.016	−.01***	−.11***	.107

^*^p <.05; **p <.01; ***p <.001; *SD *standard deviation, *IQR* interquartile range

Table 2 shows sociodemographic characteristics by PSQI category (good sleep: ≤ 5, 57.4%; poor sleep: > 5, 42.6%). No age differences occurred; however, poor sleepers had a higher household asset score (*p* = 0.033), level of education (*p* < 0.001), and were more often single (*p* = 0.03).

**Table 2:** Comparison of sociodemographic characteristics stratified by sleep quality category (Good Sleep Quality: ≤ 5 PSQI Score; Poor Sleep Quality: > 5 PSQI score)

Variable	Total (*n* = 7182)	Poor sleep quality (*n* = 3060)	Good sleep quality (*n* = 4122)	*p*
Sociodemographic factors				
Age (mean, SD), *n* = 7182	22.7 (2.8)	22.7 (2.9)	22.6 (2.8)	.285
Household asset score (mean, SD), *n* = 6876	8 (2.1)	8 (2.1)	7.9 (2.1)	.033
Level of education, *n* = 6902				<.001
< Secondary school	2471 (35.8)	979 (33.2)	1492 (37.7)	
≥ Secondary school	4431 (64.2)	1967 (66.8)	2464 (62.3)	
Relationship status, *n* = 6909				.030
Single	3110 (45)	1371 (46.5)	1739 (43.9)	
Committed relationship/married	3799 (55)	1576 (53.5)	2223 (56.1)	

*SD* standard deviation

Table 3 presents internal consistency and item-total statistics for the seven PSQI components. The internal consistency of the PSQI in this sample was acceptable (*α* = 0.57; *ω* = 0.57). Item-total correlations ranged from 0.33 (sleep medication) to 0.65 (sleep duration). Similarly, sleep medication demonstrated the weakest relationship with the remaining components (0.15), while sleep duration (0.39) and sleep efficiency (0.33) showed the strongest. Average inter-item covariances were low (0.09–0.14), indicating minimal redundancy among components and that the items capture related but distinct aspects of sleep quality, consistent with multidimensionality. Alpha-if-item-deleted ranged between 0.49 and 0.57, indicating that removal of the sleep medication component did not substantially affect the internal consistency of the PSQI. Sleep duration and sleep efficiency demonstrated stronger associations with the global score, demonstrating their alignment with the overall construct measured by the scale.

**Table 3:** Internal consistency and item-total statistics for the Pittsburgh Sleep Quality Index (PSQI) components

Component	Item-test correlation	Item-rest correlation	Average inter-item covariance	Cronbach’s alpha if item deleted	McDonald’s ω if item deleted
Subjective sleep quality	.57	.34	.11	.52	.50
Sleep latency	.53	.25	.11	.55	.54
Sleep duration	.65	.39	.09	.49	.50
Sleep efficiency	.61	.33	.10	.52	.52
Sleep disturbance	.47	.31	.12	.53	.52
Sleep medication	.33	.15	.14	.57	.58
Daytime dysfunction	.50	.30	.12	.53	.52
PSQI global score			.11	.57	.57

**Fig. 1:**
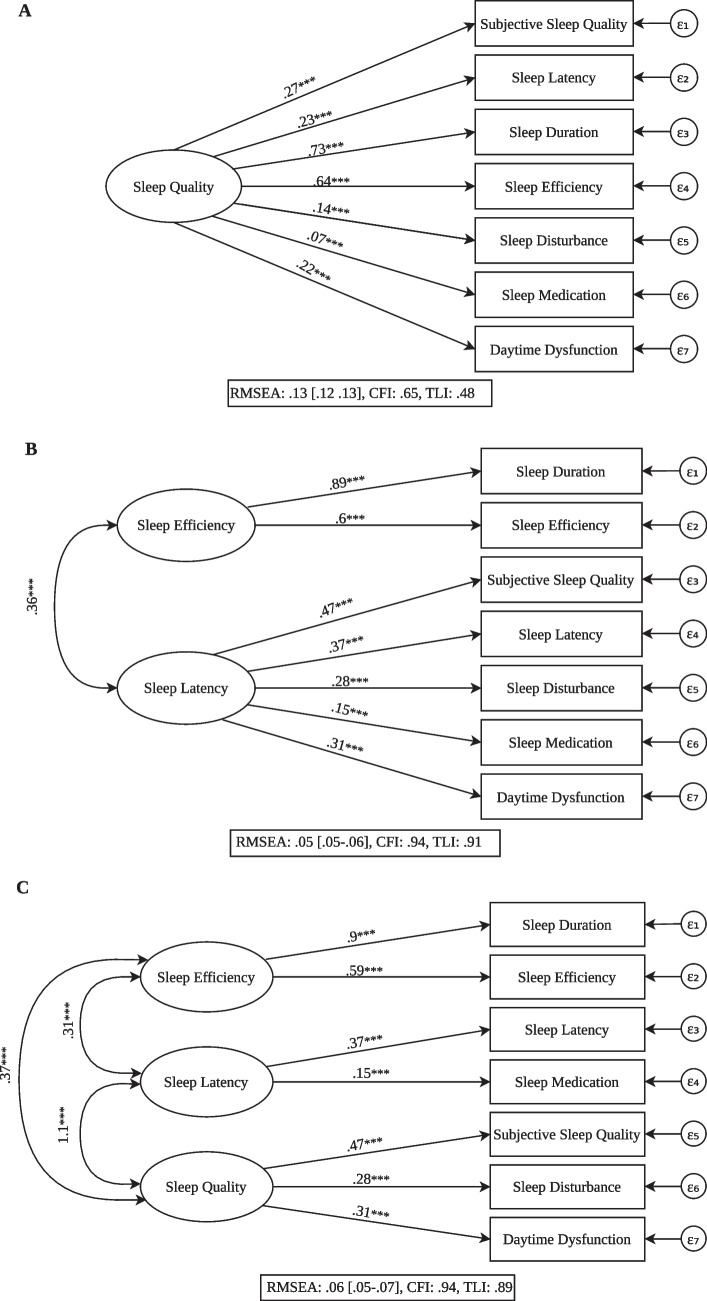
Confirmatory factor analysis models of the Pittsburgh Sleep Quality Index (PSQI): comparison of one-factor (**A**), two-factor (**B**), and three-factor structures (**C**). *RMSEA* Root Mean Square Error of Approximation; *CFI* Comparative Fit Index; *TLI* Tucker-Lewis Index. *p <.05; **p <.01; ***p <.001

Figure 1 illustrates the three-factor models. The one-factor model specified all seven PSQI components as indicators of a single latent construct, reflecting sleep quality. This model demonstrated poor fit to the data (*χ*^2^(14) = 1677.70; CFI = 0.65; TLI = 0.48; RMSEA = 0.13, 90% CI [0.12, 0.13]), indicating that a single global factor did not adequately represent the data. Both the two- and three-factor models showed improvement in fit. The two-factor model grouped the components sleep duration and sleep efficiency under the latent factor sleep efficiency, and the remaining components under sleep latency. This model showed good model fit (χ^2^(13) = 291.46; CFI = 0.94; TLI = 0.91; RMSEA = 0.05, 90% CI [0.05, 0.06]), and the two latent factors were moderately correlated (*r* = 0.36, *p* < 0.001), suggesting related but distinct dimensions of sleep. The three-factor model separated the components into sleep efficiency (sleep duration and sleep efficiency), sleep latency (sleep latency and sleep medication), and sleep quality (subjective sleep quality, sleep disturbance, and daytime dysfunction). This model demonstrated acceptable model fit (*χ*^2^(11) = 285.21; CFI = 0.94; TLI = 0.89; RMSEA = 0.06, 90% CI [0.05, 0.07]). Sleep efficiency was correlated with sleep latency (*r* = 0.31, *p* < 0.001) and sleep quality (*r* = 0.37, *p* < 0.001). However, the correlation between the sleep latency and sleep quality factors exceeded unity (*r* = 1.10, *p* < 0.001), indicating poor discriminant validity between these constructs. Therefore, the three-factor solution should be interpreted with caution, as the sleep latency and sleep quality factors may not represent distinct factors.

Table 4 presents the summary of the model fit indices for the three models. Both multifactor models demonstrated good fit to the data. Although the two- and three-factor model showed acceptable model fit, the two-factor model demonstrated slightly better model fit and did not exhibit the discriminant validity concerns observed in the three-factor model, indicating that that the PSQI reflects multiple related aspects of sleep rather than a single overall construct.

**Table 4:** Model fit comparison across models

Model	Chi-square (df)	CFI	TLI	RMSEA [90% CI]
One-factor	1677.70 (14)	.65	.48	.13 [.12–.13]
Two-factor	291.46 (13)	.94	.91	.05 [.05–.06]
Three-factor	285.21 (11)	.94	.89	.06 [.05–.07]

*df* degrees of freedom, *CFI* Comparative Fit Index, *TLI* Tucker-Lewis Index, *RMSEA* Root Mean Square Error of Approximation

## Discussion

### Key findings

Using a large sample, this study contributes to the limited psychometric evidence on the PSQI in Africa and demonstrates that among young South African women living in under-resourced urban environments, the instrument does not operate as a single, global measure of sleep quality. The one-factor model showed poor fit and modest internal consistency. While the two- and three-factor models met the accepted goodness-of-fit criteria, the two-factor model had a lower RMSEA and greater parsimony, suggesting that this model offers a more efficient and interpretable representation of sleep quality in this population that clusters into efficiency and latency. By relating factor structure and the PSQI dimensionality to participants’ environmental and psychosocial conditions, this study provides contextually informed interpretation of sleep quality rather than focusing solely on statistical model fit.

### Contextualization of findings

The prevalence of poor sleep quality in our sample (42.6%) exceeded that reported in high-income settings like Germany (36%; mean age 56.8y, 52% women) [40], but was comparable to estimates from Spain (48.7%; 18–94 years, 49–61% women) [41] and South Korea (41%; mean age = 55.3 years, 56% women; higher in women at 46.2%) [42], and less than low-resource settings (66.5%; young-middle-aged, 52% women) [43], and South African HIV patients (61%; mean age = 42.7 years, 79% women) [28]. Differences in prevalence highlight the influence of contextual adversity, such as environmental, psychosocial, and health-related stress, supported by sociodemographic findings in this study. Higher education, lower household assets, and single status were linked to poorer sleep, likely from combined educational or employment pressures, low-SES stressors (e.g., unhealthy behaviors, chronic conditions), and reduced social support [44–48]. No age effects likely reflected our young sample [43, 49]. However, these findings should be interpreted with caution as poor sleep quality was determined using the global PSQI cutoff score despite this study demonstrating limited support of the unidimensional structure.

Although the PSQI was originally designed as a single global score measure, a large body of literature suggests that sleep quality is better conceptualized as a multidimensional construct. An influential validation of this perspective was presented by Cole et al. [50], who proposed a three-factor model in older adults. This framework has subsequently informed PSQI scoring and interpretation across different populations and aligns with Buysse’s conceptualization of sleep quality as comprising multiple, interrelated domains [51].

Similarly, our results demonstrated the PSQI does not exhibit a single latent structure, as both the two- and three-factor models provided a better representation of the data. While these findings contradict evidence from high- and low-income countries, namely Canada and Sri Lanka, respectively [16, 17], they correspond with a broader body of literature. In particular, support of the three-factor model has been reported among older adults in Portugal [22], Iranian student samples [21], Korean clinical populations [52] and pregnant women in Peru [53]. At the same time, evidence from other populations supports a two-factor structure, including pregnant women in the USA [54], female workers in New Zealand during COVID-19 lockdown conditions [19], and multinational student samples from Africa, Southeast Asia and South America [20]. However, even within this multinational study, differences in factor structures occurred across subsamples, with the Peruvian subgroup supporting a three-factor model [20], suggesting that the PSQI dimensionality may be influenced by contextual or population-related factors.

While both multidimensional models showed comparable performance, the two-factor model was preferred in this study due to its greater parsimony, marginally improved model fit, and better conceptual clarity in this setting. The first factor corresponds to the sleep efficiency domain, which captures objective aspects of sleep, such as duration and proportion of time spent asleep while in bed. The second factor integrates components separated into the sleep quality indicator of the three-factor model (i.e., subjective sleep quality, sleep disturbance, and daytime dysfunction). Selecting the two-factor model does not imply that the three-factor model is invalid, rather, it suggests that in our sample, domains that are distinct in other contexts are possibly experienced as overlapping aspects of a shared, underlying experience and, therefore, combine into fewer factors. As such, for young women in Soweto, nocturnal disturbances, subjective evaluations of sleep, and daytime fatigue appear to reflect a single pattern of fragmented sleep. Consequently, sleep problems in this setting may not arise from insufficient opportunity to sleep, but from an inability to achieve continuous, uninterrupted sleep despite adequate time in bed.

This explanation is supported by the standardized factor loadings. Sleep duration and efficiency consistently showed a high loading across the multidimensional models, suggesting these aspects are readily distinguishable as they are more objective and easier to conceptualize. In contrast, sleep disturbance, daytime dysfunction and sleep medication were below the accepted 0.40 threshold [55]. The weaker loadings for sleep latency, sleep disturbance, and daytime dysfunction possibly indicate that participants do not experience these as separate domains but rather as an overlapping aspect of sleep disruption. These loadings are possibly due to the experiential and subjective nature of the items, which are influenced by mood, stress and environmental factors. In contrast, the consistently low loading of sleep medication use across all models likely reflects contextual factors, including limitations in the availability and affordability of prescription medications [56, 57] and a lack of awareness that poor sleep quality is a concern requiring further treatment [28]. In urban South African townships, women often experience significant environmental and psychosocial stressors, such as material deprivation, overcrowded households, caregiving demands and fear-driven hypervigilance that may disrupt sleep onset, reduce sleep efficiency, and normalize fatigue [58–60]. These contextual factors may explain why the PSQI did not function as a single unified construct in this population and instead produced multidimensional representations of sleep reflecting: (1) behavioral sleep efficiency, capturing more objective, time-based elements of sleep, and (2) perceived sleep quality, shaped by chronic stress and environmental unpredictability. Under such conditions, women may not clearly separate particular aspects of sleep, but instead experience them as a combined experience. Nevertheless, identifying the specific causes of these disturbances or clarifying how participants interpreted individual PSQI items fell beyond the scope of the present psychometric analysis and warrants further investigation.

### Implications

This study evaluated the PSQI among young South African women and shows that the global score does not adequately capture sleep quality in this context. While both the two- and three-factor models provide better model fit than the unidimensional model, the two-factor model provided a simpler and clearer explanation of the data. Our findings highlight the need for context-specific psychometric validation when applying standardized sleep instruments across diverse populations and contribute to international information on the PSQI’s psychometric properties and the importance of modelling overlapping, time-based components when examining sleep constructs.

Clinically, the results suggest that screening and intervention should focus on specific PSQI components (i.e., nocturnal disruptions and environmental factors) rather than relying on the global score. Interventions for young South African women may also require targeting stress-related sleep disorders, environmental disturbances, and perceived sleep quality rather than focusing only on sleep duration.

Finally, the study highlights sleep as a social determinant of health for young women in low-resource settings, likely influenced by safety, overcrowding, caregiving demands, and financial stress. Integrating sleep health into community, women’s health, and public policy initiatives may strengthen overall wellbeing.

### Limitations and future research

Although our study benefited from a large, community-based sample, the cross-sectional design limits information on how sleep patterns change over time and the inclusion of only young, urban women from the Soweto region limits the extent to which findings can be generalized to men, older adults, and individuals living in rural settings. Various PSQI components demonstrated weak factor loadings, which suggests that particular sleep domains may not function as distinct constructs in this population. However, the focus of this study was not to adjust models to improve fit but rather compare established models while retaining their intended structure. The use of self-report measures also introduces potential reporting biases, particularly for sleep duration and daytime dysfunction, and increases the risk of participants misunderstanding or misinterpreting PSQI items. Since no qualitative interviewing accompanied the survey, we cannot determine how respondents interpreted PSQI items or conceptualized key constructs such as “sleep quality.” Finally, the data violated assumptions of multivariate normality, which may affect parameter estimates in some model specifications, underscoring the need for analytic approaches that account for ordinal and non-normally distributed data.

Future research should evaluate whether the PSQI measures sleep in the same way for different groups of people in the South African context, to ensure the factor structure is valid and comparable across the population. Longitudinal studies are needed to clarify bidirectional relationships between PSQI domains and mental health, caregiving burden, and socioeconomic-related stress. Qualitative research will provide valuable insight into how participants interpret PSQI items and define “good sleep,” thereby informing culturally appropriate adaptations to the scale. In addition, future research could extend this analysis by using ordinal estimators and alternative model specifications to explore whether these approaches further enhance construct validity in diverse South African populations.

## Conclusion

Our findings indicate that, while the PSQI single global sleep-quality score should be interpreted with caution in this under-resourced urban South African cohort, the instrument remains appropriate when using an empirically supported multidimensional (component/factor-informed) scoring approach. The preference for a parsimonious two‑factor model highlights the importance of distinguishing between behavioral sleep efficiency and perceived sleep quality, rather than relying on a global score, in order to better capture sleep continuity and disrupted sleep experiences. We therefore recommend integrating PSQI-based sleep quality assessment into African research studies and clinical trials, to strengthen women’s health frameworks and enable evaluation of interventions targeting the social and behavioral determinants of health.

## Supplementary Information

Below is the link to the electronic supplementary material.

ESM 1 (DOCX 32.5 KB)

## Data Availability

The datasets generated and/or analyzed during the current study are available from the corresponding author on request.
