# Supplementary material for: Psychometric evaluation of the Pittsburgh Sleep Quality Index among South African women participating in the Bukhali trial: Healthy Life Trajectories Initiative
Source: J Clin Sleep Med. 2026 Aug 3;22(1):131. doi: 10.1007/s44470-026-00146-z (PMC13433665; doi:10.1007/s44470-026-00146-z)
Supplement: Supplementary file 1 [file 44470_2026_146_MOESM1_ESM.docx]

**PSQI SUPPLEMENTARY MATERIAL**

**Table 1.**

*Descriptives of the Comparison of Pittsburgh Sleep Quality Index (PSQI) Items and Components (n =7182)*

|  | **n (%)** | **Mean (SD)** | **Median (IQR)** | **Skewness** | **Kurtosis** |
| --- | --- | --- | --- | --- | --- |
| PSQI Global Score |  | 5.4 (3.1) | 5 (3 – 7) | .77 | 3.5 |
| ***Item*** |  |  |  |  |  |
| During the past month, what time have you usually gone to bed at night? (hours, adjusted for midnight crossing) |  | 21.63 (2) | 22 (21-22.5) | -2.29 | 17.1 |
| During the past month, how long has it usually taken you to fall asleep each night? (mins) |  | 25.3 (24.5) | 20 (10 -30) | 2.47 | 12.68 |
| During the past month, what time have you usually gotten up in the morning? (hrs) |  | 7.15 (1.7) | 7 (6-8) | -.45 | 12.11 |
| During the past month, how many hours of actual sleep did you get at night? (hrs) |  | 7.81 (1.88) | 8 (7-9) | .09 | 3.36 |
| During the past month, how often have you had trouble sleeping because you… |  |  |  |  |  |
| Cannot get to sleep within 30 minutes |  |  |  |  |  |
| Not during the past month | 2967 (41.3) |  |  |  |  |
| Less than once a week | 955 (13.3) |  |  |  |  |
| Once or twice a week | 1218 (17) |  |  |  |  |
| Three or more times a week | 2042 (28.4) |  |  |  |  |
| Wake up in the middle of the night or early morning |  |  |  |  |  |
| Not during the past month | 2160 (30.1) |  |  |  |  |
| Less than once a week | 1164 (16.2) |  |  |  |  |
| Once or twice a week | 1794 (25) |  |  |  |  |
| Three or more times a week | 2064 (28.7) |  |  |  |  |
| Have to get up to use the bathroom |  |  |  |  |  |
| Not during the past month | 2605 (36.3) |  |  |  |  |
| Less than once a week | 781 (10.9) |  |  |  |  |
| Once or twice a week | 1304 (18.1) |  |  |  |  |
| Three or more times a week | 2492 (34.7) |  |  |  |  |
| Cannot breathe comfortably |  |  |  |  |  |
| Not during the past month | 5809 (80.9) |  |  |  |  |
| Less than once a week | 523 (7.3) |  |  |  |  |
| Once or twice a week | 501 (7) |  |  |  |  |
| Three or more times a week | 349 (4.8) |  |  |  |  |
| Cough or snore loudly |  |  |  |  |  |
| Not during the past month | 5462 (76.1) |  |  |  |  |
| Less than once a week | 569 (7.9) |  |  |  |  |
| Once or twice a week | 530 (7.4) |  |  |  |  |
| Three or more times a week | 621 (8.6) |  |  |  |  |
| Feel too cold |  |  |  |  |  |
| Not during the past month | 4601 (64.1) |  |  |  |  |
| Less than once a week | 922 (12.8) |  |  |  |  |
| Once or twice a week | 905 (12.6) |  |  |  |  |
| Three or more times a week | 754 (10.5) |  |  |  |  |
| Feel too hot |  |  |  |  |  |
| Not during the past month | 4814 (67) |  |  |  |  |
| Less than once a week | 821 (11.4) |  |  |  |  |
| Once or twice a week | 852 (11.9) |  |  |  |  |
| Three or more times a week | 695 (9.7) |  |  |  |  |
| Have bad dreams |  |  |  |  |  |
| Not during the past month | 4344 (60.5) |  |  |  |  |
| Less than once a week | 1028 (14.3) |  |  |  |  |
| Once or twice a week | 1050 (14.6) |  |  |  |  |
| Three or more times a week | 760 (10.6) |  |  |  |  |
| Have pain |  |  |  |  |  |
| Not during the past month | 5188 (72.2) |  |  |  |  |
| Less than once a week | 694 (9.7) |  |  |  |  |
| Once or twice a week | 705 (9.8) |  |  |  |  |
| Three or more times a week | 595 (8.3) |  |  |  |  |
| Other |  |  |  |  |  |
| Not during the past month | 5955 (82.9) |  |  |  |  |
| Less than once a week | 362 (5.1) |  |  |  |  |
| Once or twice a week | 397 (5.5) |  |  |  |  |
| Three or more times a week | 468 (6.5) |  |  |  |  |
| During the past month, how would you rate your sleep quality overall? |  |  |  |  |  |
| Very Good | 2927 (40.8) |  |  |  |  |
| Fairly Good | 3091 (43) |  |  |  |  |
| Fairly Bad | 712 (9.9) |  |  |  |  |
| Very Bad | 452 (6.3) |  |  |  |  |
| During the past month, how often have you taken medicine to help you sleep (prescribed or “over the counter”)? |  |  |  |  |  |
| Not during the past month | 6491 (90.4) |  |  |  |  |
| Less than once a week | 308 (4.3) |  |  |  |  |
| Once or twice a week | 243 (3.4) |  |  |  |  |
| Three or more times a week | 140 (1.9) |  |  |  |  |
| During the past month, how often have you had trouble staying awake while driving, eating meals, or engaging in social activity? |  |  |  |  |  |
| Not during the past month | 5952 (82.9) |  |  |  |  |
| Less than once a week | 649 (9) |  |  |  |  |
| Once or twice a week | 406 (5.7) |  |  |  |  |
| Three or more times a week | 175 (2.4) |  |  |  |  |
| During the past month, how much of a problem has it been for you to keep up enough enthusiasm to get things done? |  |  |  |  |  |
| No problem at all | 5168 (72) |  |  |  |  |
| Only a slight problem | 1335 (18.6) |  |  |  |  |
| Somewhat a problem | 390 (5.4) |  |  |  |  |
| A very big problem | 289 (4) |  |  |  |  |
| ***Component*** |  |  |  |  |  |
| Subjective Sleep Quality |  |  |  |  |  |
| 0 | 2927 (40.8) |  |  |  |  |
| 1 | 3091 (43) |  |  |  |  |
| 2 | 712 (9.9) |  |  |  |  |
| 3 | 452 (6.3) |  |  |  |  |
| Sleep Latency |  |  |  |  |  |
| 0 | 1894 (26.4) |  |  |  |  |
| 1 | 2453 (34.1) |  |  |  |  |
| 2 | 1896 (26.4) |  |  |  |  |
| 3 | 939 (13.1) |  |  |  |  |
| Sleep Duration |  |  |  |  |  |
| 0 | 4195 (58.4) |  |  |  |  |
| 1 | 1355 (18.9) |  |  |  |  |
| 2 | 884 (12.3) |  |  |  |  |
| 3 | 748 (10.4) |  |  |  |  |
| Sleep Efficiency |  |  |  |  |  |
| 0 | 4251 (59.2) |  |  |  |  |
| 1 | 1467 (20.4) |  |  |  |  |
| 2 | 662 (9.2) |  |  |  |  |
| 3 | 802 (11.2) |  |  |  |  |
| Sleep Disturbance |  |  |  |  |  |
| 0 | 514 (7.1) |  |  |  |  |
| 1 | 4817 (67.1) |  |  |  |  |
| 2 | 1746 (24.3) |  |  |  |  |
| 3 | 105 (1.5) |  |  |  |  |
| Sleep Medication |  |  |  |  |  |
| 0 | 6491 (90.4) |  |  |  |  |
| 1 | 308 (4.3) |  |  |  |  |
| 2 | 243 (3.4) |  |  |  |  |
| 3 | 140 (1.9) |  |  |  |  |
| Daytime Dysfunction |  |  |  |  |  |
| 0 | 4600 (64) |  |  |  |  |
| 1 | 1915 (26.7) |  |  |  |  |
| 2 | 564 (7.9) |  |  |  |  |
| 3 | 103 (1.4) |  |  |  |  |
| hrs = hours; min =minutes; SD = standard deviation; IQR = interquartile range. | | | | | |
